# MLST and RAPD molecular analysis of *Staphylococcus aureus* subsp. *anaerobius* isolated from goats in Poland

**DOI:** 10.1007/s00203-018-1568-1

**Published:** 2018-09-04

**Authors:** Olga Szaluś-Jordanow, Katarzyna Krysztopa-Grzybowska, Michał Czopowicz, Agata Moroz, Marcin Mickiewicz, Anna Lutyńska, Jarosław Kaba, Tomasz Nalbert, Tadeusz Frymus

**Affiliations:** 1 Division of Infectious Diseases, Department of Small Animal Diseases with Clinic, Warsaw University of Life Sciences-SGGW, Nowoursynowska 159c Street, 02-776 Warsaw, Poland; 2 Department of Sera and Vaccine Evaluation, National Institute of Public Health-National Institute of Hygiene (NIPH-NIH), 24 Chocimska Street, 00-791 Warsaw, Poland; 3 Laboratory of Veterinary Epidemiology and Economics, Warsaw University of Life Sciences-SGGW, Nowoursynowska 159 Street, 02-776 Warsaw, Poland; 4 Department of Medical Biology, The Cardinal Stefan Wyszyński Institute of Cardiology, Alpejska 42 Street, 04-628 Warsaw, Poland

**Keywords:** *Staphylococcus aureus* subsp. *anaerobius*, MLST, RAPD, Goat, Morel’s disease

## Abstract

*Staphylococcus aureus s*ubsp. *anaerobius* is an etiological agent of Morel’s disease in small ruminants. The infection results in superficial abscesses located near lymph nodes. In the study, molecular analysis based on multilocus sequence typing (MLST) of seven housekeeping genes (*arcC, aroE, glp, gmk, pta, tpi, yqiL*) and random amplified polymorphic DNA (RAPD) was carried out on 19 *S. aureus* subsp. *anaerobius* strains isolated from two different goat herds from Poland. All of the 19 *S. aureus* subsp. *anaerobius* strains were found to belong to single MLST and RAPD types which support the high clonality level of this agent. However, the results obtained show clearly that the *S. aureus* subsp. *anaerobius* clone found in goats in Poland is different from those previously described. However, it is identical to the ATCC 38844 strain isolated from sheep in Spain, which has not been so far genotyped using MLST.

## Introduction and aim

*Staphylococcus aureus* subsp. *anaerobius* is a Gram-positive, coccus-shaped bacterium growing typically at 37 °C on 5% sheep blood-enriched Columbia agar in microaerophilic conditions (de la Fuente et al. 1985). *S. aureus* subsp. *anaerobius* causes Morel’s disease in small ruminants, inducing subcutaneous abscesses measuring form several to 30 cm (Szaluś-Jordanow et al. 2010). Clinical signs are observed in young animals, mostly at the age of less than 6 months and rarely in adult ones (Møller et al. 2000). Morel’s disease in goats is regarded as a rare or frequently misdiagnosed condition. The disease has been described in many countries, inter alia: Somali (Pegram 1973), Sudan (el Sanousi et al. 1989), Kenya (Shirlaw and Ashford 1962), Saudi Arabia (Alhendi et al. 1993), Italy (Valenti and Bielar 1984), Croatia (Habrun et al. 2004) and Poland (Szaluś-Jordanow et al. 2010). Availability of *S. aureus* subsp. *anaerobius*-sequenced genomes allows to apply molecular tools that might precisely differentiate this infection from that caused by atypical *S. aureus* subsp. *aureus* or other catalase-negative staphylococci (Elbir et al. 2013). Different molecular methods, such as genotyping, have been applied in inexpensive and fast tracking of the strain genetic similarity during epidemiological studies. Random amplified polymorphic DNA (RAPD) technique allowed to reveal genetic differences within many bacterial species (Gzyl and Augustynowicz 1999), however, had not been used before for genotyping of *S. aureus* subsp. *anaerobius*. The aim of this study was to perform a genetic analysis of *S. aureus* subsp. *anaerobius* strains isolated from goats in Poland using multilocus sequence typing (MLST) and compare its discriminatory potential to that of RAPD.

## Materials and methods

In the study, molecular analysis was carried out on 19 *S. aureus* subsp. *anaerobius* strains isolated from two goat herds from Poland. According to the information obtained from the goat’s owner, transfer between the herds was made in the past. Reference strains of *S. aureus* subsp. *anaerobius* ATCC 35844 and *S. aureus* subsp. *aureus* ATCC 29213 bought directly from ATCC via http://www.lgcstandards.com/atcc were used in the study. According to the https://www.lgcstandards-atcc.org/products/all/35844.aspx?geo_country=pl#documentation, ATCC 35844 strain was isolated from a young sheep in Spain (de la Fuente et al. 1985).

Strains were cultured on Columbia agar with 5% sheep blood and incubated for 48 h at 37 °C in aerobic and microaerophilic conditions. Chromosomal DNA was isolated using commercially available kit modified by the inclusion of lysostaphin (SIGMA).

MLST of seven housekeeping genes: carbamate kinase (*arcC*), shikimate dehydrogenase (*aroE*), glycerol kinase (*glp*), guanylate kinase (*gmk*), phosphate acetyltransferase (*pta*), triosephosphate isomerase (*tpi*), and acetyl coenzyme A acetyltransferase (*yqiL*) were performed according to Enright et al., 2000 with small modifications. PCR was carried out with 20-µl reaction volumes containing 20 ng of DNA, 20 pmol of each primer and 10 µl of JumpStart REDTaq ReadyMix PCR Reaction Mix (SIGMA), and performed with an initial denaturation (95 °C/5 min) followed by 30 cycles of denaturation (95 °C/30 s), annealing (55 °C/30 s), extension (72 °C/45 s) followed by a final extension step (72 °C/5 min). Sequencing of both strands was performed by external service (Genomed S.A.). Analysis of the sequencing results and classification of MLST types was performed using the database available at https://pubmlst.org/saureus/.

Random amplified polymorphic DNA reaction was performed using Ready-To-Go RAPD analysis beads (GE Healthcare Life Sciences). Each bead containing buffer, dNTPs, bovine serum albumin and thermostable DNA polymerase was mixed with 20 ng of DNA, 25 pmol of Primer 6 (5′-d[CCCGTCAGCA]-3′) and H_2_O. PCRs were performed with an initial denaturation (95 °C/5 min) followed by 45 cycles of denaturation (95 °C/1 min), annealing (36 °C/1 min), extension (72 °C/2 min) followed by a final extension step (72 °C/2 min). RAPD profiles were analyzed using BioNumerics Software (6.6, Applied Maths) according to the UPGMA algorithm with the Pearson’s coefficient.

## Results

**Fig. 1:**
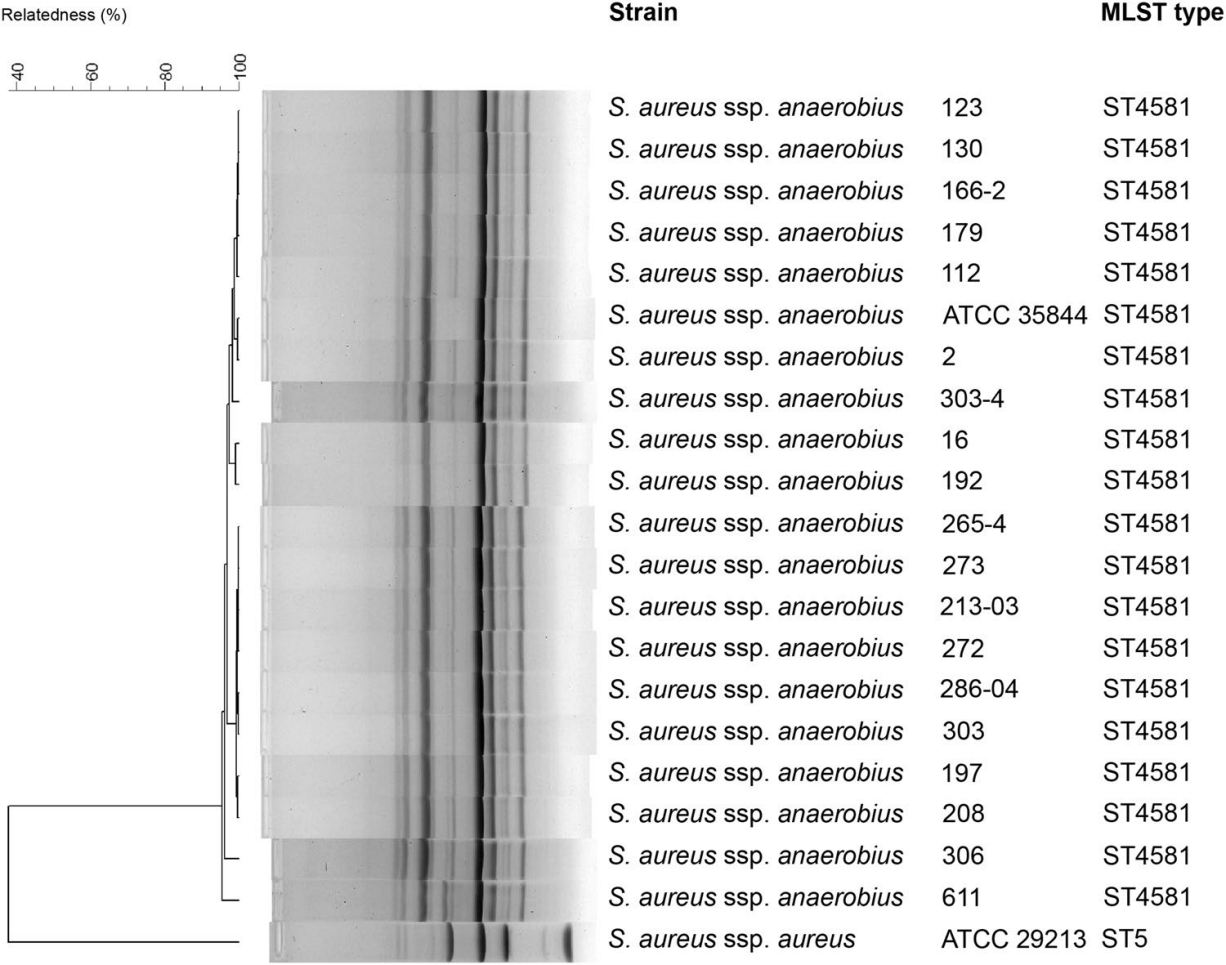
RAPD and MLST analysis of the 19 clinical *S. aureus* ssp. *anaerobius* isolates and two reference strains

All 19 *S. aureus* subsp. *anaerobius* strains and the ATCC 35844 strain were found to be single MLST and RAPD profile types. RAPD analysis showed > 95% genetic similarity among the tested isolates (Fig. 1). According to the MLST database, all strains were assigned as harboring the MLST-type ST4581 (102, 219, 204, 122,13, 422, 502) that differs by three substitutions in the *tpi* and *yqiL* genes from the widely described in the literature ST1464 type (Fig. 1). Analysis of sequences available in the https://pubmlst.org/saureus/ database revealed high similarity of our MLST type to the ST3756 type isolated from sheep in the Czech Republic, as they differ only by a single nucleotide polymorphism in the *pta* gene.

## Discussion and conclusions

Several publications from other countries suggested high genetic homogeneity of *S. aureus* subsp. *anaerobius* isolates. Musa et al. (2010) found this in the sequences of catalase-encoding gene as they identified only a single mutation typical for African strains in comparison with European isolates. European and African strains have been found to share 13–15 mutations in the *kat* gene, and these subtle differences were regarded by de la Fuente et al. (2011) as related to the host adaptation. Elbir et al. (2013) found all 17 caprine *S. aureus* subsp. *anaerobius* strains from Sudan to harbor a single MLST-type ST1464, confirming high subspecies clonality. Similarly, MLST data obtained by de la Fuente et al. (2011) for 94 strains of *S. aureus* subsp. *anaerobius* isolated from small ruminants in Spain (*n* = 79), Italy (*n* = 9), Denmark (*n* = 3) and Sudan (*n* = 3) confirmed high genetic homogeneity as seen by PFGE and sharing of the same ST1464 type of MLST. In our study, we found that the goats in two Polish herds were infected with a single *S. aureus* subsp. *anaerobius* MLST clone assigned as ST4581 (Fig. 1). This MLST type is very similar to ST1464, commonly found in strains isolated worldwide, differing only by three point mutations. Furthermore, ST4581 found in our goats differs from a strain isolated in the Czech Republic by only one point mutation in the *pta* gene (ST3756). The Czech Republic is the neighbor country of Poland, with a border in the mountains, and exchange of small ruminants between flock owners could have taken place in the past. Further studies on whole genome sequences of strains originating from Poland and the Czech Republic might elucidate the character of changes in the view of the thesis of country specific host adaptation of isolates involved in Morel disease in small ruminants.

In conclusion, our finding supports previous findings about high genetic homogeneity of *S. aureus* subsp. *anaerobius* isolates. The MLST clone ST4581 variations found in this study have not been described elsewhere. However, it is identical to the ATCC 38844 strain isolated from a sheep in Spain. Thus far, this reference strain has not been genotyped with MLST method. As seen in Fig. 1, both MLST and RAPD were capable to differentiate the *aureus* and *anaerobius* substrains of *S. aureus*. As we described before (Szaluś-Jordanow et al. 2010), one animal with asymptomatic infection is sufficient to introduce the disease into a herd. In our case it was a male goat bought from Germany. It is possible that Morel’s disease is widespread in Europe, however, remains misdiagnosed for caseous lymphadenitis caused by *Corynebacterium pseudotuberculosis*. In Germany, where the male goat came from, Morel’s disease had not been even described.

Abscess disease was additionally confirmed to be a clonally spread as genetic similarity of analysed strains measured by RAPD fingerprinting was almost identical which is consistent with earlier studies (Szaluś-Jordanow et al. 2011, 2013).
